# Education, Healthy Ageing and Vaccine Literacy

**DOI:** 10.1007/s12603-021-1627-1

**Published:** 2021-04-10

**Authors:** Jean-Pierre Michel, J. Goldberg

**Affiliations:** 1 Honorary professor of Medicine, Medical University of Geneva, Geneva, Switzerland; 2 Honorary professor of Medicine, Medical University 1 of Lyon, Lyon, France

**Keywords:** Education, health literacy, vaccine literacy, vaccine empowerment, vaccine uptake, life course

## Abstract

**Importance and Objective:**

The Covid pandemic is a timely opportunity to try to broaden our understanding of the links between education and health literacy and explore the vaccine-decision process with a view to identifying interventions that will positively influence vaccine uptake.

**Evidence:**

Health and vaccine literacy encompass people’s knowledge, motivation, and competence to access, understand, appraise and apply health information in order to make judgements and take decisions in everyday life concerning health care, disease prevention and health promotion.

**Findings:**

Appropriate vaccine communication, which depends greatly on personal and contextual determinants, as well as on societal and environmental circumstances, is essential to reassure people about vaccine efficacy, safety, and possible side effects. However, vaccine confidence is not solely a question of trust in the vaccine’s efficacy, safety. and individual protective benefit of vaccination. It also encompasses the mechanism(s) of vaccine activity, immunization schedules, organization and trust in the healthcare system that promotes and delivers the vaccines, and at what costs.

When healthcare professionals as science brokers of vaccine knowledge attempt to increase vaccine knowledge and confidence, they must adjust their communication to the educational or health literacy level of their intended audience. Even if their messages are apparently clear and simple, they absolutely need to verify that they are properly understood.

**Relevance:**

Specific vaccine communication training appears essential to increase vaccine communication skills among healthcare providers. Moreover, further randomized controlled studies are warranted to improve vaccine empowerment among different populations, from a variety of educational backgrounds.

**T**he recent SARS-2-CoV pandemic has totally changed the relationships between healthcare systems and people, moving from the older “provider-centered” and more recent “patient-centered” models of care delivery, to a new model that has emerged spontaneously, namely a “people-centered” approach, corresponding to the “co-creation of health”. This emerging concept includes the understanding of epidemiological data, public health and cluster surveillance, transmission pathways and hygiene. Individual physical or social distancing, preventive or so-called “barrier” gestures, hand-washing, wearing of masks, and restricting movements have all become the daily rules propounded to protect ourselves, our children and older adults from “THE virus”. The pandemic also highlights more than ever before the importance of risk factors, the limits on access to healthcare systems, and the difficulties involved in the development, safety and accreditation of new drugs and vaccines (1, 2). Moreover, never in human history has there been such an abundance of health information available from so many (more or less trustworthy) sources (3).

In parallel, researchers in multiple domains have been applying their knowledge to try to correlate pandemic epidemiology with recent evaluations of health literacy in a range of European countries (4–7). The results prompted Dirk Van Damme, senior consultant at the OECD to argue that there may be a correlation between education level and pandemic severity (8).

This complex context is a timely opportunity to try to broaden our understanding of the links between education and health literacy, and to analyse the existing connections with vaccine literacy. Subsequently, it would also be of interest to explore the vaccine-decision process with a view to identifying interventions that will positively influence vaccine uptake.

## Basic education and adult learning vs. health literacy

It is well established that the relationship between education and health may be confounded by childhood socioeconomic circumstances and/or cognitive ability, and may be explained by several major factors, including material conditions, social-psychological resources and a healthy lifestyle (9). In most countries around the world, education has an impact on life expectancy. For example, in France, the partial (35 to 80 years) life expectancy of low, moderate, and highly educated men varies from 37.6 years, to 39.5 and 41.3 years respectively (10). These large, education-related differences in life expectancy integrate the effects of unhealthy life conditions or habits, with factors such as low income, high body mass index and smoking accounting for gaps of 8.3%, 10.2% and 23% respectively in the partial life expectancy of low-educated men compared to highly-educated men (10). Education inequity also impacts life expectancy via the accumulation of risk factors, poor self-reported health (11), inadequate chronic disease management and disability (12). Indeed, it appears easy to link basic education and adult learning with health literacy, i.e. the process of enabling people to increase their control over, and improve their own health (13). Health literacy encompasses people’s knowledge, motivation and competence to access, understand, appraise and apply health information in order to make judgements and take decisions in everyday life concerning health care, disease prevention and health promotion, to maintain or improve quality of life (14, 15). This chain of tasks (information, comprehensibility, meaningfulness, and manageability) depends greatly on personal and contextual determinants, as well as on societal and environmental circumstances (15).

In 2015, for the first time, a consortium from 8 countries of the European Union (EU) reported findings from the European Health Literacy Survey (HLS-EU), which comprises 47 items across 12 subdomains, and enables comparison of health literacy levels between member states (16). Among the 8 countries included in the analysis, excellent health literacy levels varied from 9.1% in Spain to 25.1% in the Netherlands, whereas 26.9% of Bulgarians were found to have inadequate health literacy, compared to only 1.8% in the Netherlands. That is to say that problematic to totally inadequate literacy fluctuates from 28.7% in the Netherlands, to 58.3% of the population in Spain and 62.1% in Bulgaria. The high rate of people with low education or/and literacy raises tremendous problems. Difficulties with reading, writing, numeracy, communication, and the use of electronic technology impede access to an adequate understanding of health care information. This is especially problematic when it comes to reading and understanding the information on package inserts provided with prescription medicine. Low health literacy competencies have also been shown to result in less knowledge about health, riskier behavior, poorer overall health, less ability to deal with chronic diseases, less self-management, more hospitalizations, and increased care costs (14, 17, 18).

## Health literacy vs. Vaccine literacy

In addition to health literacy, vaccine literacy, which requires the individual to seek out relevant information among the ever-increasing data glut in the media (particularly on the Internet) and make an appropriate decision about vaccination. Vaccine literacy can increase vaccine uptake (19). An exploratory quantitative study from Hong Kong perfectly illustrates the relationships that exist between health literacy and vaccine practices. Among 486 community dwelling older adults aged over 65 (60% of whom had low basic education), health literacy was evaluated by the Chinese version of the European Health Literacy Survey (HLS-Asia-Q) (20). The HLS-Asia-Q findings revealed a high rate of problematic or inadequate health literacy in particular in the domains relating to finding information (83.5%), understanding information (77.4%), evaluating information (84.1%) and applying information (63.9%) (20). The consequences of deficient health literacy perfectly explain the participants’ inability to find vaccine information, interpret it, and appraise it, resulting in increased difficulty in making vaccination decisions (20). Generally, people with low education have low self-reported global health, and their main questions regarding vaccines relate to the duration of the vaccine protection and the cost to themselves (19). Conversely, people with a high educational level, who do not always have high self-reported global health, are more concerned by the vaccine efficacy, possible vaccine side effects and costs (19).

These different types of questions raise different communication issues. Very often, the messages relayed by the mass media and healthcare professionals are too complex, or not properly understood, or not clear enough to be convincing regarding the vaccine’s usefulness. This is particularly true in the context of the communication dilemma whereby, on the one hand, we seek to prompt citizens to exert their autonomy and make well-informed vaccine decisions that are relevant to their own health, while on the other hand, we impose mandatory vaccination programmes to increase the level of herd immunity in the population (21).

**Figure 1:**
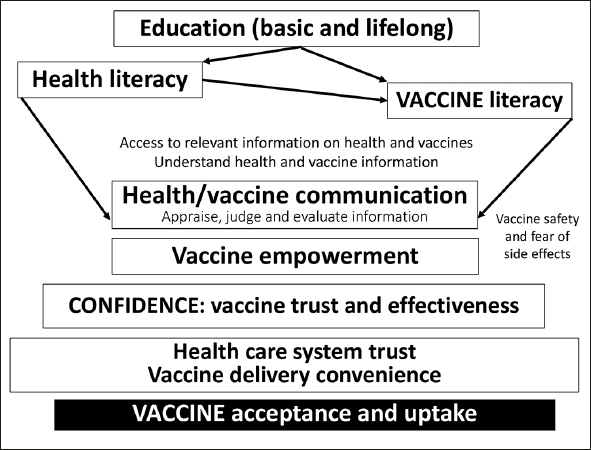
The vaccine decision process which is mainly based on health and vaccine literacy includes vaccine comprehensibility, meaningfulness, and manageability. The vaccine acceptance and uptake depend on each stage of this complex decision process (Figure independently created)

## The vaccine decision

Appropriate vaccine communication is essential to reassure people about vaccine efficacy, safety, and possible side effects, while simultaneously underlining the individual protective benefit of vaccination. One of the most important steps for any healthcare professional seeking to empower parents or adults is to verify whether the vaccine information has been clearly understood. If not, information adjustment may be needed, with re-assessment of the message comprehensibility, which is essential to instilling vaccine confidence. However, vaccine confidence is not solely a question of trust in the vaccine’s efficacy and safety, but also encompasses the mechanism(s) of vaccine activity, immunization schedules, organization and trust in the healthcare system that promotes and delivers the vaccines (19), and at what costs. Vaccine confidence and convenience, which are the results of a very complex and dynamic process in a context of many outlier or fringe opinions, are essential steps on the path to vaccine acceptance and uptake (22). Regardless, it appears that the empowerment process can lead to disparate results, depending on the population concerned: young adults under 40 years of age reject influenza (flu) vaccination, while adults over 65 are more inclined to be in favour of flu vaccination, but against pneumococcal vaccination. Such discrepancies are frequent, despite the constant pleas of healthcare professionals in favour of vaccine acceptance and uptake.

## Examples of vaccine empowerment of the population

With such an imbroglio of basic education, health, and vaccine literacy, it is challenging to determine which interventions can best favour vaccine uptake, to the benefit of the whole population. Nevertheless, three examples involving different vaccines and target populations are presented below.

The first example is a US, nationally representative, web-based survey, to promote measles-mumps-rubella (MMR) vaccination. A total of 1759 parents with at least one child under 17 years of age in their household, were randomly assigned to either a control group, or to receive one of the following four interventions: (1) correcting misinformation about vaccine-related autism; (2) presenting information about the disease risks; (3) displaying visuals of a child with measles; or (4) a dramatic narrative about infant who almost died from the disease. The results were disappointing. The correction of misinformation and visuals of sick children decreased the fear of vaccine-related autism, the dramatic narrative increased the fear of MMR vaccine-related side effects, while providing information about the diseases prevented by the vaccine had no effect. Overall, these interventions aimed at reducing misperceptions about MMR vaccine modified neither vaccine confidence nor vaccine uptake (23).

The second illustrative example is a pragmatic, cluster-randomized crossover trial in 22 private general practitioners clinics in Singapore, and involved 4378 and 4459 patients over 65 years of age who visited the clinics during the intervention and control periods, respectively (24). Two time periods were analysed, separated by a one-month wash-out: the intervention period, lasting 3 months, during which posters and flyers on influenza and pneumococcal diseases and vaccines were made available; and a control period. Comparison of the intervention vs control periods showed an increase in flu vaccine uptake (5.9% vs 4.8%, p=0.047), and in pneumococcal vaccine uptake (5.7% vs 3.7%, p=0.001), largely drive by patients suffering from diabetes, hypertension, and/or dyslipidaemia (24).

Finally, the third example was a study involving a pharmacist-drive programme to promote pneumococcal vaccination (“Pharmacists Pneumonia Prevention Program”, PPPP) (25). A total of 190 cognitively intact volunteers (mean age 74.4 years, 80.5% African-American; 76.3% females) participated in 1.5-hour information session including a pharmacist-led presentation, actors’ skit, and small-group action planning. The results were extremely positive, with a 46.3% increase in knowledge about the risks of pneumococcal disease, transmission, symptoms, and vaccine side-effects after the intervention, and a 54.2% increase in knowledge recall at 3 month post-intervention, in comparison to baseline knowledge levels. Moreover, the overall increase in pneumococcal vaccine uptake reached 37.2% in previously unvaccinated participants. These very encouraging results were somewhat offset by the individual cost of each session, which amounted to 119 USD per participant (25).

**Figure 2:**
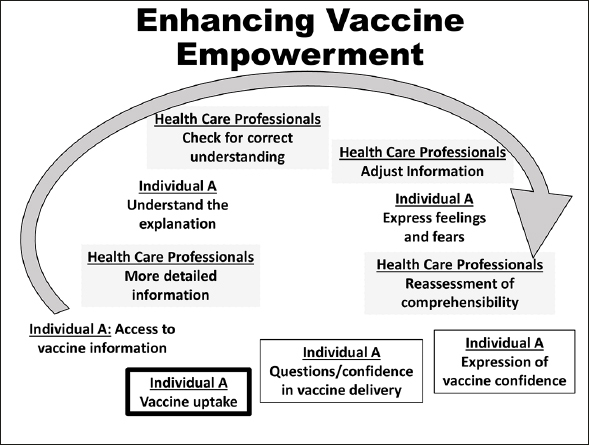
Enhancing vaccine empowerment (Figure independently created based in concepts from WITINK H et al — 18). The vaccine empowerment depends greatly on consumers-healthcare professionals communication, which relies on providers’ vaccine knowledge and communication skills allowing better consumers ‘trust in vaccine efficacy/safety in vaccine delivery

These three examples of web-based training, simple flyers, posters and leaflets, and face-to-face meetings aimed at improving knowledge of infectious diseases and vaccine among populations with varying levels of education, clearly demonstrate the need to select the appropriate message, which in turn must be accurate and phrased so as to be clearly understood. Controlling the comprehensibility of the information and its reassessment are probably the key to the success of the Pharmacists Pneumonia Prevention Program (25).

## Take-home messages

- When attempting to increase vaccine knowledge and confidence, do not neglect to adapt your communication to the educational or health literacy level of your intended audience. Give a clear and simple message and verify that it is properly understood.
- The role of healthcare professionals as brokers of vaccine knowledge is fundamental. Therefore, specific vaccine communication training is essential to increase vaccine communication skills among healthcare providers.
- Further randomized controlled studies are warranted to improve vaccine empowerment among different populations, from a variety of educational backgrounds, and to better identify the optimal strategies for improving vaccine uptake.

## Notes

*Ethical standards:* None

## References

[1] Lazcano-Ponce E, Alpuche-Aranda C (2020). Public health literacy in the face of the Covid-19 pandemic emergency. Salud Publica Mex.

[2] OECD (2019). Health for Everyone? Social Inequalities in Health and Health Systems.

[3] Abel T, McQueen D. Critical health literacy and the COVID-19 crisis. Health Promot Int. 2020. 10.1093/heapro/daaa040 PMC7184450 32239213

[4] Lopes H, McKay V. Adult learning and education as a tool to contain pandemics: The COVID-19 experience. Int Rev Educ. 2020:1–28. 10.1007/s11159-020-09843-0 PMC7302500 32836371

[5] Spring H. Health literacy and COVID-19. Health Info Libr J. 2020. 10.1111/hir.12322 PMC7405264 32672399

[6] OECD (2019). Skills Matter. Additional Results from the Survey of Adult Skills.

[7] Paakkari L, Okan O (2020). COVID-19: health literacy is an underestimated problem. Lancet Public Health.

[8] van Damme D. The School of Life: Do levels of education and skills influence the health risks of COVID-19? Available at: https://www.oecd-forum.org/posts/the-school-of-life-do-levels-of-education-and-skills-influence-the-health-risks-of-covid-19 [Access date: 3 September 2020]. 2020.

[9] Kulhanova I, Hoffmann R, Judge K, Looman CW, Eikemo TA, Bopp M (2014). Assessing the potential impact of increased participation in higher education on mortality: evidence from 21 European populations. Soc Sci Med.

[10] Mackenbach JP, Valverde JR, Bopp M, Bronnum-Hansen H, Deboosere P, Kalediene R (2019). Determinants of inequalities in life expectancy: an international comparative study of eight risk factors. Lancet Public Health.

[11] Sole-Auro A, Martin U, Dominguez Rodriguez A. Educational Inequalities in Life and Healthy Life Expectancies among the 50-Plus in Spain. Int J Environ Res Public Health. 2020;17. 10.3390/ijerph17103558 PMC7277913 32438706

[12] Maki N, Martikainen P, Eikemo T, Menvielle G, Lundberg O, Ostergren O (2013). Educational differences in disability-free life expectancy: a comparative study of longstanding activity limitation in eight European countries. Soc Sci Med.

[13] World Health Organization, editor. The Ottawa Charter for Health Promotion. Available at: https://www.who.int/healthpromotion/conferences/previous/ottawa/en/1986.

[14] World Health Organization (2013). Health Literacy: The solid facts.

[15] Sorensen K, Van den Broucke S, Fullam J, Doyle G, Pelikan J, Slonska Z (2012). Health literacy and public health: a systematic review and integration of definitions and models. BMC Public Health.

[16] Sorensen K, Pelikan JM, Rothlin F, Ganahl K, Slonska Z, Doyle G (2015). Health literacy in Europe: comparative results of the European health literacy survey (HLS-EU). Eur J Public Health.

[17] van der Heide I, Wang J, Droomers M, Spreeuwenberg P, Rademakers J, Uiters E (2013). The relationship between health, education, and health literacy: results from the Dutch Adult Literacy and Life Skills Survey. J Health Commun.

[18] Wittink H, Oosterhaven J (2018). Patient education and health literacy. Musculoskelet Sci Pract.

[19] Lorini C, Santomauro F, Donzellini M, Capecchi L, Bechini A, Boccalini S (2018). Health literacy and vaccination: A systematic review. Hum Vaccin Immunother.

[20] Zhang F, Or PP, Chung JW (2020). The effects of health literacy in influenza vaccination competencies among community-dwelling older adults in Hong Kong. BMC Geriatr.

[21] Veldwijk J, van der Heide I, Rademakers J, Schuit AJ, de Wit GA, Uiters E (2015). Preferences for Vaccination: Does Health Literacy Make a Difference?. Med Decis Making.

[22] Danchin MH, Costa-Pinto J, Attwell K, Willaby H, Wiley K, Hoq M (2018). Vaccine decision-making begins in pregnancy: Correlation between vaccine concerns, intentions and maternal vaccination with subsequent childhood vaccine uptake. Vaccine.

[23] Nyhan B, Reifler J, Richey S, Freed GL (2014). Effective messages in vaccine promotion: a randomized trial. Pediatrics.

[24] Ho HJ, Tan YR, Cook AR, Koh G, Tham TY, Anwar E (2019). Increasing Influenza and Pneumococcal Vaccination Uptake in Seniors Using Point-of-Care Informational Interventions in Primary Care in Singapore: A Pragmatic, Cluster-Randomized Crossover Trial. Am J Public Health.

[25] Pizzi LT, Prioli KM, Fields Harris L, Cannon-Dang E, Marthol-Clark M, Alcusky M (2018). Knowledge, Activation, and Costs of the Pharmacists’ Pneumonia Prevention Program (PPPP): A Novel Senior Center Model to Promote Vaccination. Ann Pharmacother.

